# Mechanism of activation of the *BNLF2a* immune evasion gene of Epstein-Barr virus by Zta

**DOI:** 10.1099/jgv.0.001056

**Published:** 2018-03-26

**Authors:** Rajaei Almohammed, Kay Osborn, Sharada Ramasubramanyan, Ijiel Barak Naranjo Perez-Fernandez, Anja Godfrey, Erika J. Mancini, Alison J. Sinclair

**Affiliations:** School of Life Sciences, University of Sussex, Brighton, East Sussex, UK; ^†^​ Present address: Centre for Gene Regulation and Expression, College of Life Sciences, University of Dundee, Dow Street, Dundee DD1 5EH, UK.; ^‡^​ Present address: RS Mehta Jain Department of Biochemistry and Cell Biology, Vision Research Foundation, Sankara Nethralaya, Chennai, India.

**Keywords:** Epstein-Barr virus, *BNLF2a*, immune evasion, gene expression, BZLF1, epigenetics

## Abstract

The human gamma herpes virus Epstein–Barr virus (EBV) exploits multiple routes to evade the cellular immune response. During the EBV lytic replication cycle, viral proteins are expressed that provide excellent targets for recognition by cytotoxic T cells. This is countered by the viral *BNLF2a* gene. In B cells during latency, where *BNLF2a* is not expressed, we show that its regulatory region is embedded in repressive chromatin. The expression of *BNLF2a* mirrors the expression of a viral lytic cycle transcriptional regulator, Zta (BZLF1, EB1, ZEBRA), in B cells and we propose that Zta plays a role in up-regulating *BNLF2a*. In cells undergoing EBV lytic replication, we identified two distinct regions of interaction of Zta with the chromatin-associated *BNLF2a* promoter. We identify five potential Zta-response elements (ZREs) in the promoter that are highly conserved between virus isolates. Zta binds to these elements *in vitro* and activates the expression of the *BNLF2a* promoter in both epithelial and B cells. We also found redundancy amongst the ZREs. The EBV genome undergoes a biphasic DNA methylation cycle during its infection cycle. One of the ZREs contains an integral CpG motif. We show that this can be DNA methylated during EBV latency and that both Zta binding and promoter activation are enhanced by its methylation. In summary, we find that the *BNLF2a* promoter is directly targeted by Zta and that DNA methylation within the proximal ZRE aids activation. The implications for regulation of this key viral gene during the reactivation of EBV from latency are discussed.

## Introduction

Epstein–Barr virus (EBV) is a human gamma herpes virus that has a lifetime association with the host and can enter into a state of long-term latency in memory B cells [1]. During viral latency, most of the EBV lytic replication cycle genes are transcriptionally silent [2–5]. A repressive chromatin context is considered to contribute to this, as some EBV lytic replication cycle promoters within the EBV genome have been shown to be associated with heterochromatin, polycomb-associated chromatin or DNA methylation (e.g. [6–12]). In response to differentiation signals, EBV undergoes the lytic replication cycle in both B cells [13] and epithelial cells [14].

The viral transcription factor Zta (BZLF1, ZEBRA, EB1) is essential for this process [15]. Zta interacts with sequence-specific DNA elements in epigenetically repressed chromatin and drives the activation of certain viral lytic replication cycle genes [16, 17]. Genome-wide mapping of Zta interactions with the viral genome revealed many further genes that are potentially directly regulated by interaction with Zta [18, 19].

Expression of the viral gene *BNLF2a* occurs during the early phase of EBV lytic cycle replication [20]. *BNLF2a* has an important role in evading immune surveillance by encoding a 60-amino acid protein that interferes with antigen presentation to CD8+ cells. This is achieved through blocking the peptide- and ATP-binding functions of transporter-associated antigen processing (TAP) [21–25]. The relevance of *BNLF2a* is highlighted by the impact that a genetic knock-out mutation of *BNLF2a* has on cells newly infected with EBV and those undergoing the lytic cycle – they become more susceptible to recognition by CD8+ T cells [22, 26]. The expression of BNLF2a mRNA and protein follows from Zta during EBV reactivation [3, 22], suggesting a coordinated mechanism of regulation or a direct link between the two.

Here we questioned how regulation of *BNLF2a* is achieved during lytic reactivation. We present evidence that the promoter is associated with repressive chromatin during latency and that it can be activated through the direct interaction of Zta with sequence-specific Zta binding elements (ZREs) in the promoter region. An unexpected redundancy between multiple functional Zta binding sites was revealed through biochemical and genetic analyses. Additionally, we find that the proximal ZRE can be subject to DNA methylation during latency and that this leads to enhanced DNA binding and activation by Zta. Conservation of these elements across virus isolates underscores the importance of fail-safe mechanisms to ensure appropriate activation of this critically important gene.

## Results

### A repressive chromatin environment surrounds the BNLF2a promoter during viral latency

**Fig. 1.**
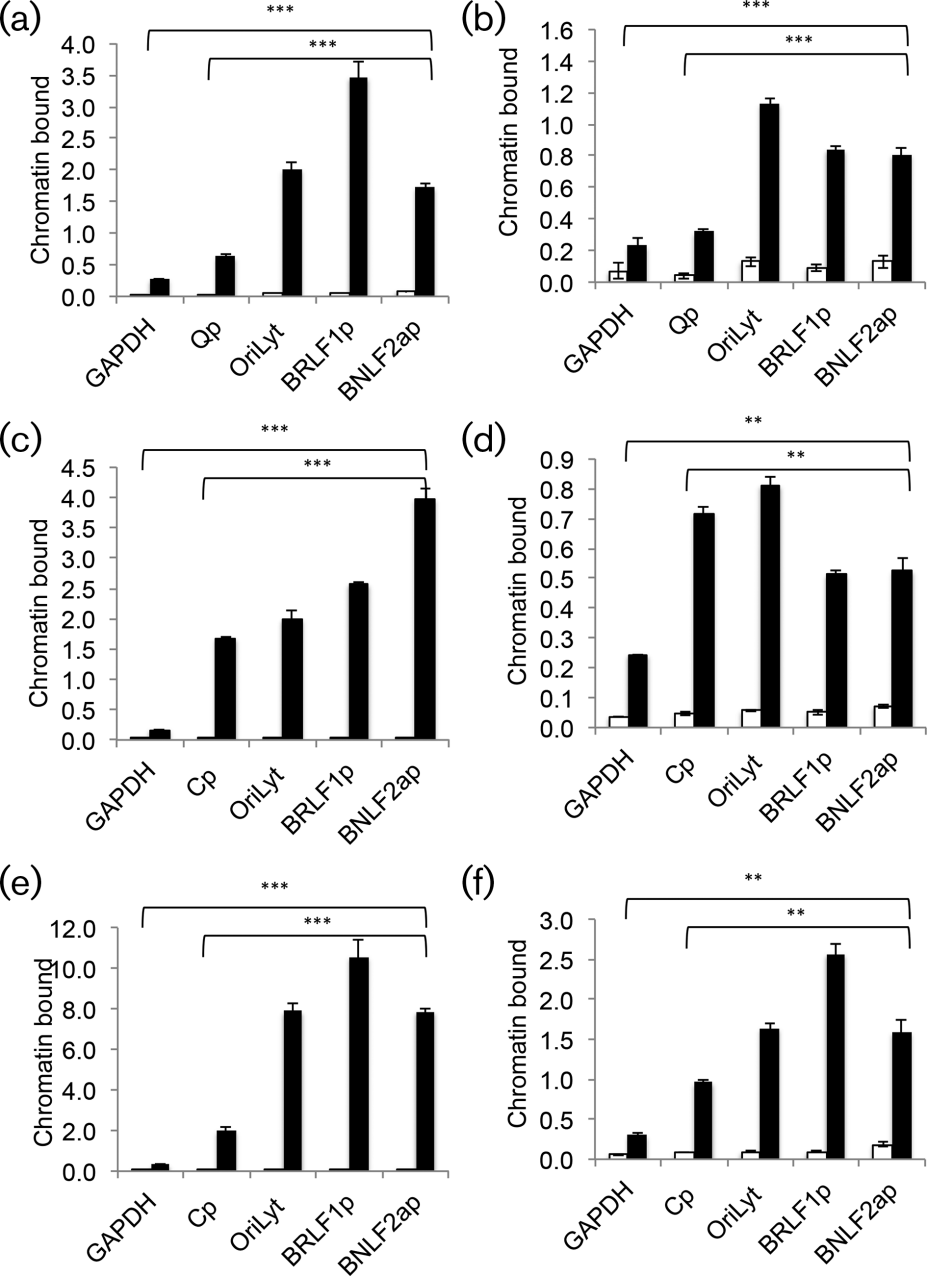
Chromatin organization at the *BNLF2a* promoter is associated with repressive H3K27me3 and H3K9me3 modifications during latency. Chromatin was isolated from cells harbouring latent EBV, an LCL (a, b), Akata BL (c, d) and Raji BL (e, f) cells. Chromatin precipitation was undertaken with antibodies specific for the modified histones (H3K27me3 (a, c, e) and H3K9me3 (b, d, f) and their relevant species-specific controls. DNA was eluted from the precipitate and the relative amounts of each of the indicated loci analysed by Q-PCR relative to the input genomes, and is expressed as a percentage of input binding. In each case the standard deviation is shown (triplicate measurements). The significance of the difference in binding is shown as **P≤0.01; ***P 0.001).

The *BNLF2a* gene is not expressed during EBV latency within B cells. We asked whether the promoter for *BNLF2a* is associated with repressive chromatin: H3K9me3, a marker of heterochromatin, or H3K27me3, a marker of polycomb repressive complexes [27]. We undertook chromatin precipitation experiments from two latent Burkitt's lymphoma (BL) cell lines (Akata and Raji) and a tightly latent lymphoblastoid cell line (GM2188). Precipitation with a control non-specific antibody was used to set the baseline for the ChIP assays. Analysis of H3K27me3 and H3K9me3 with three EBV lytic cycle-associated loci (OriLyt, the BRLF1 promoter and the BNLF2a promoter) and two active promoters (GAPHD and either a latency promoter [Qp (Akata) or Cp (Raji and LCL)], revealed a significant enrichment of H3K27me3 and H3K9me3 with the *BNLF2a* promoter for each cell type, compared to the control antibody (*P*≤0.01) (Fig. 1). Furthermore, the association with the BNLF2a promoter was significantly increased compared to association with two active promoters in each cell line (*P*≤0.01). The pattern of enrichment for markers of repressive chromatin at latency/lytic regions of EBV genomes within the three different cell types conforms to the general pattern seen in other cells [7, 8, 28, 29], as do data from tightly latent LCLs within the Encode database [30].

### Zta interacts with the BNLF2a promoter in cells

The Zta transcription factor plays a central role in activating the expression of many EBV genes [31]. Expression of both Zta and *BNLF2a* is activated during EBV lytic replication [3, 22]. This prompted us to ask whether *BNLF2a* might be a direct transcriptional target of Zta.

**Fig. 2.**
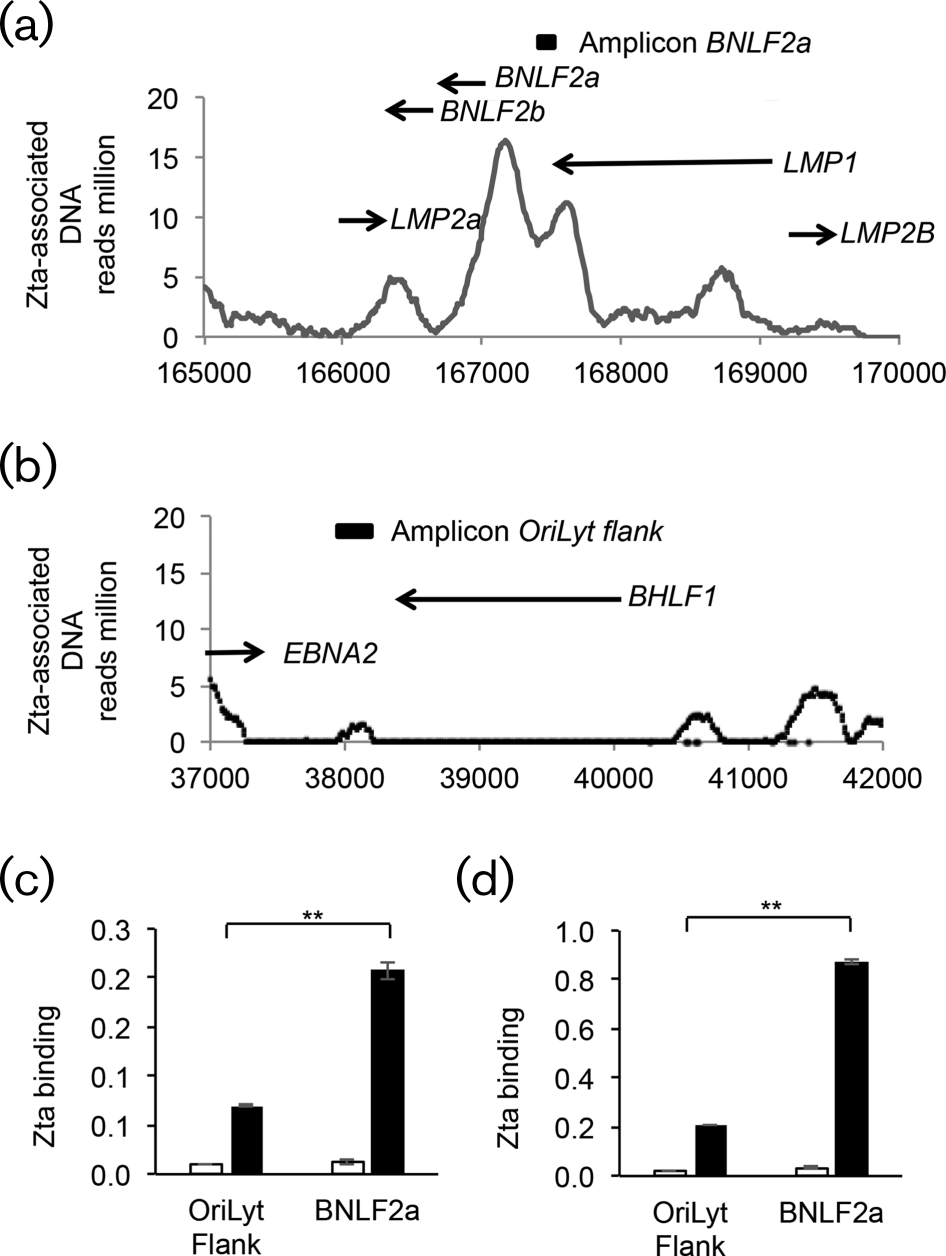
Zta binding to the *BNLF2a* promoter region. ChIP-Seq data from Akata BL cells undergoing lytic cycle with an antibody that recognizes chromatin-bound Zta was aligned the EBV genome. The nucleotide position of the EBV genome is shown on the x-axis. The locations of gene coding regions are shown as arrows, with amplicons used to identify the specific regions by ChIP shown as boxes. (a). The *BNLF2a* Locus. (b). The OriLyt region. (c-d). ChIP coupled to Q-PCR analysis of Zta binding was undertaken from chromatin from cells harbouring lytic EBV: induced Akata BL cells (48 h) (c) and spontaneously lytic LCL#3 cells (d). The Zta ChIP is shown as a black bar and the control antibody as an open bar. Q-PCR amplification of the indicated loci was undertaken in triplicate. The axes show percentage binding relative to input chromatin and the error bars indicate the standard deviation. The significance of the difference in Zta binding to OriLyt flank and Zta is shown (**P≤0.01).

A genome-wide chromatin immunoprecipitation (ChIP) dataset detailing the interaction of Zta with the EBV genome in Akata cells undergoing the lytic replication cycle (induced by stimulation with anti-IgG for 48 h) [19] was mined (Fig. 2a, b). The relative signal for input chromatin is compared in Fig. S1(a, b, available in the online version of this article). In this experiment, the average size of Zta-associated peaks was 316±244 nucleotides. The analysis identified binding sites for Zta on the EBV genome in the *BNLF2a* promoter region (Fig. 2a); the signal was specific to the Zta ChIP data track (Fig. S1). A region of the genome 2 Kb distal to Orilyt, which showed low binding by Zta, was identified as a control low binding region (Fig. 2b). To confirm Zta binding and to question whether a similar association with Zta occurs in other cells, we undertook further chromatin immunoprecipitation from two cell lineages where EBV undergoes a lytic replication cycle: a group I BL cell line (Akata) (induced by stimulation with a low dose of anti-IgG for 48 h) and a spontaneously lytic lymphoblastoid cell line (LCL#3) [32]. Both cell populations showed an equivalent level of lytic cells as determined by intracellular staining and FACs analysis (6–7 % Zta-positive data not shown). We identified significant enrichment of Zta at the *BNLF2a* promoter compared to a region flanking OriLyt in both cell types (*P*≤0.01) (Fig. 2c, d).

### Conservation of the BNLF2a promoter

To question whether the association of Zta with the *BNLF2a* promoter is likely to be direct or indirect, we used a pattern-matching ZRE prediction tool that we previously validated [33]. This identified five potential ZREs within the *BNLF2a* promoter: a cluster of two (distal) and a cluster of three (proximal) (Fig. S2). The DNA sequences of the five ZREs were used to generate a position weight matrix that strongly resembles that found for Zta interaction with DNA in genome-wide Zta-association studies [18, 34] (Fig. S2). We then compared the conservation of the *BNLF2a* promoter sequence among 92 isolates of EBV (Table S1) to ask whether these elements are conserved. This revealed a high degree of conservation of the integrity and location of the ZREs (ZREs 1–4 100% and ZRE5 86 %) (Fig. S2).

### Zta interaction with the BNLF2a promoter

**Fig. 3.**
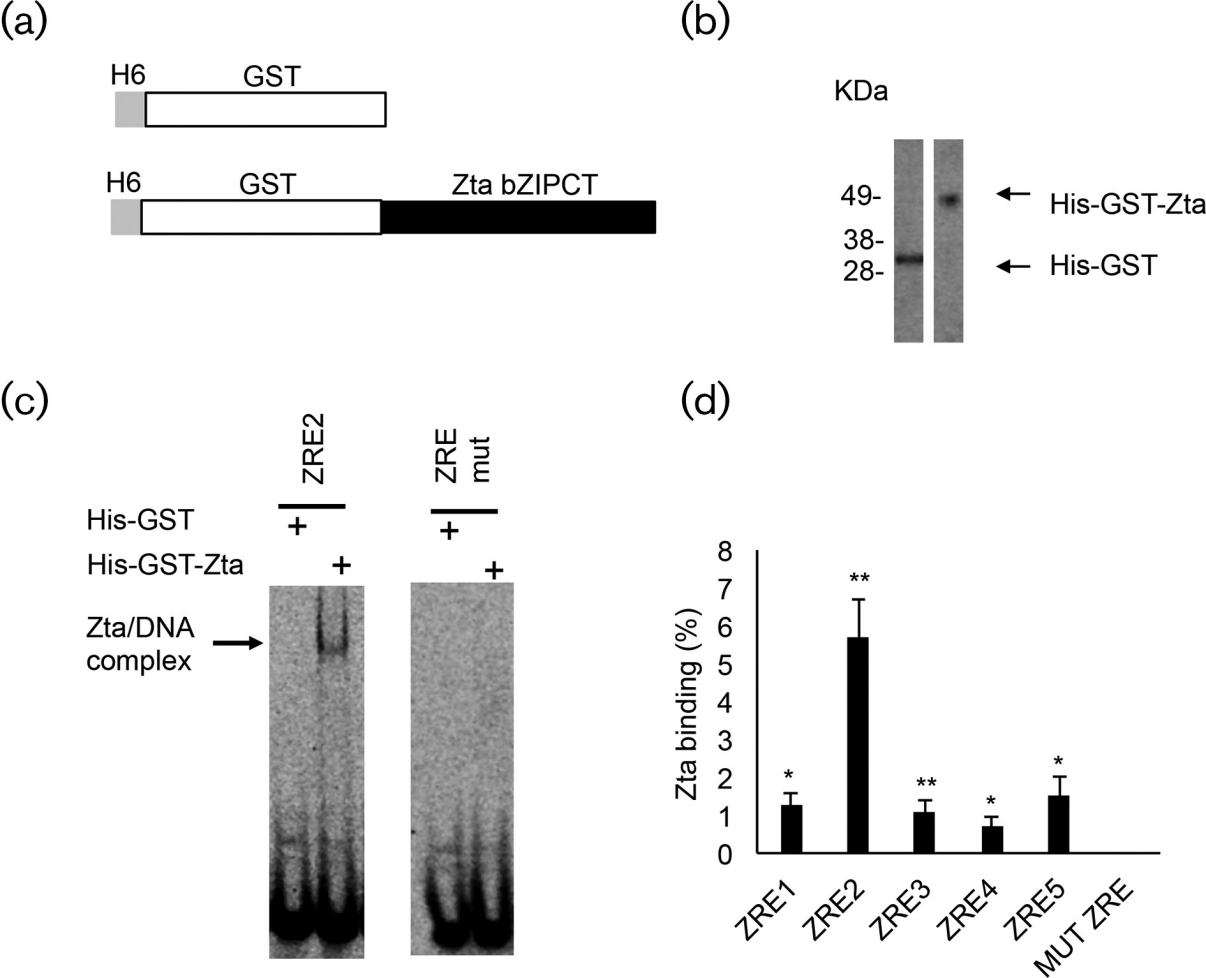
Interaction between Zta and the *BNLF2a* ZREs *in vitro.* (a). A His-tagged GST-Zta expression vector encoding the DNA binding and dimerization region of Zta was generated. (b). Protein was produced in *E. coli* and purified using cobalt ion affinity purification. 160 ng of the resulting protein was fractionated on SDS-PAGE together with 160 ng of a his-tagged GST protein and visualized with Simply Blue stain. (c). 80 ng of His GST and His-GST-Zta proteins were incubated with IR-labelled double-strand oligonucleotide probes corresponding to BNLF2a ZRE2 or a mutant version of the sequence, and the reactions were then separated on native polyacrylamide gels using EMSA. The migration of free DNA and bound DNA complexes is shown. (d). Equivalent EMSAs were undertaken with each of the individual BNLF2a ZREs and the mutant version. The bar graph shows quantitation of binding (% of probe bound) with the standard deviation from triplicate assays. The statistical significance of binding was compared to the signal generated with His-GST protein (*P≤0.05; **P≥0.01).

To ask whether Zta binds directly to any of the ZREs, we cloned, expressed and purified a His-tagged GST-Zta fusion protein (amino acids 168–245), containing the DNA binding and dimerization region of Zta that has been used previously to address DNA binding specificity (e.g. [18, 35]) (Fig. 3a, b). IR-labelled double-strand oligonucleotides corresponding to each of the five ZREs were used to identify any interaction of Zta with site binding using electrophoretic mobility shift assays (Fig. 3c). His-GST protein was employed as control to identify potential background levels of DNA retardation, and showed negligible binding to the ZREs compared to his-GST-Zta. This is shown for ZRE2 in Fig. 3(c). As a further control, a version of ZRE2 with mutations in the ZRE was constructed and evaluated for binding by His-GST or His-GST-Zta. Negligible binding was observed (Fig. 3c). We then questioned the ability of His-GST-Zta to bind with each of the five *BNLF2a* ZREs (Figs 3d and S3). This revealed that his-GST-Zta interacted significantly (*P*≤0.05) with each of the five ZREs, with the highest binding observed at ZRE2 (Fig. 3d).

### Activation of the BNLF2a promoter through ZREs

**Fig. 4.**
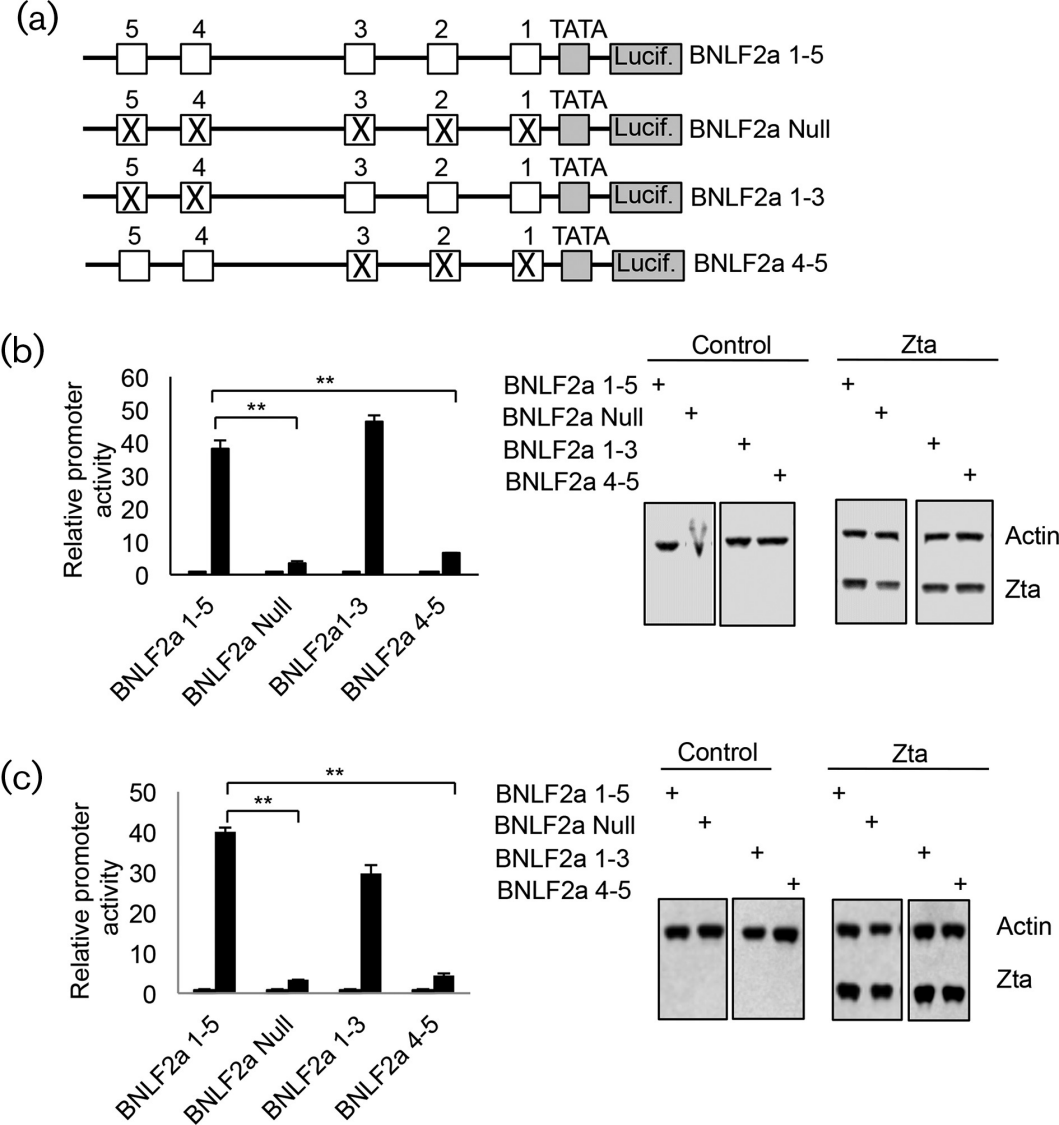
Contribution of Zta-binding regions to *BNLF2a* promoter activation by Zta. (a). Schematic diagrams of the BNLF2a promoter–luciferase reporter system. The ZREs are represented by 1–5, with the TATA box and start of the luciferase gene shown. The mutation of ZREs is represented by an X. (b). The indicated plasmids were introduced into DG75 cells with the his-Zta expression vector (black box) or a control plasmid (open box). Cells were incubated for 48 h and luciferase activity determined. Zta and actin protein expression were determined following Western blotting. The statistical significance of the difference in Zta-driven promoter activity between BNLF2a 1–5 and the mutants is shown **P≤0.01). (c). The indicated plasmids were introduced into HeLa cells with either the his-Zta expression vector or a control plasmid. Cells were incubated for 48 h and Zta-driven promoter activity determined as in (b).

In order to determine whether Zta is able to drive activation of the *BNLF2a* promoter, we cloned a region around the transcription start site of the BNLF2a gene into a luciferase reporter vector (BNLF2a 1–5) (Figs S2, S5 and 4a). We then introduced this into cells that do not contain EBV: a BL cell line DG75 [36] and the HeLa epithelial cell line [37]. Co-transfection with Zta drove induction of BNLF2a 1–5 in both cell types (Fig. 4b, c). In DG75, Zta increased gene expression by 38-fold and in HeLa cells Zta increased expression by 40-fold. In both cases mutation of all five ZREs (BNLF2a Null) lowered baseline activity (Fig. S4a, b) and caused a dramatic and significant reduction in Zta-mediated induction (*P*≤0.01). In each case there was only a small (1.3-fold) difference in transfection efficiency (as monitored by Zta protein expression). These results show that the majority of Zta-mediated promoter activation occurs through the ZREs.

Multiple mutations were then introduced into the ZREs within each of the two Zta ChIP regions, resulting in simultaneous mutation of all three ZREs within the proximal Zta ChIP region and leaving the distal ZREs intact (pBNLF2a 4–5), or in mutations in both of the distal ZREs, leaving the proximal region intact (pBNLF2a 1–3) (Fig. 4). In both cases a small variation in basal promoter activity was detected (Fig. S4). The ability of Zta to activate these promoters was compared. Mutating the distal Zta ChIP site (BNLF2a 1–3) resulted in non-significant changes in Zta activation in both cell types. However, a significant (*P*≤0.01) reduction in Zta activation was found as a result of mutating the proximal Zta ChIP site in both cell types (BNLF2a 4–5). A 6-fold (DG75 cells) and 10-fold (HeLa cells) reduction in Zta activation of BNLF2a 4–5 by Zta was observed. In these experiments, only small differences in transfection efficiency of between 1.5- and 1.6-fold were evident, as monitored by Zta protein abundance.

**Fig. 5.**
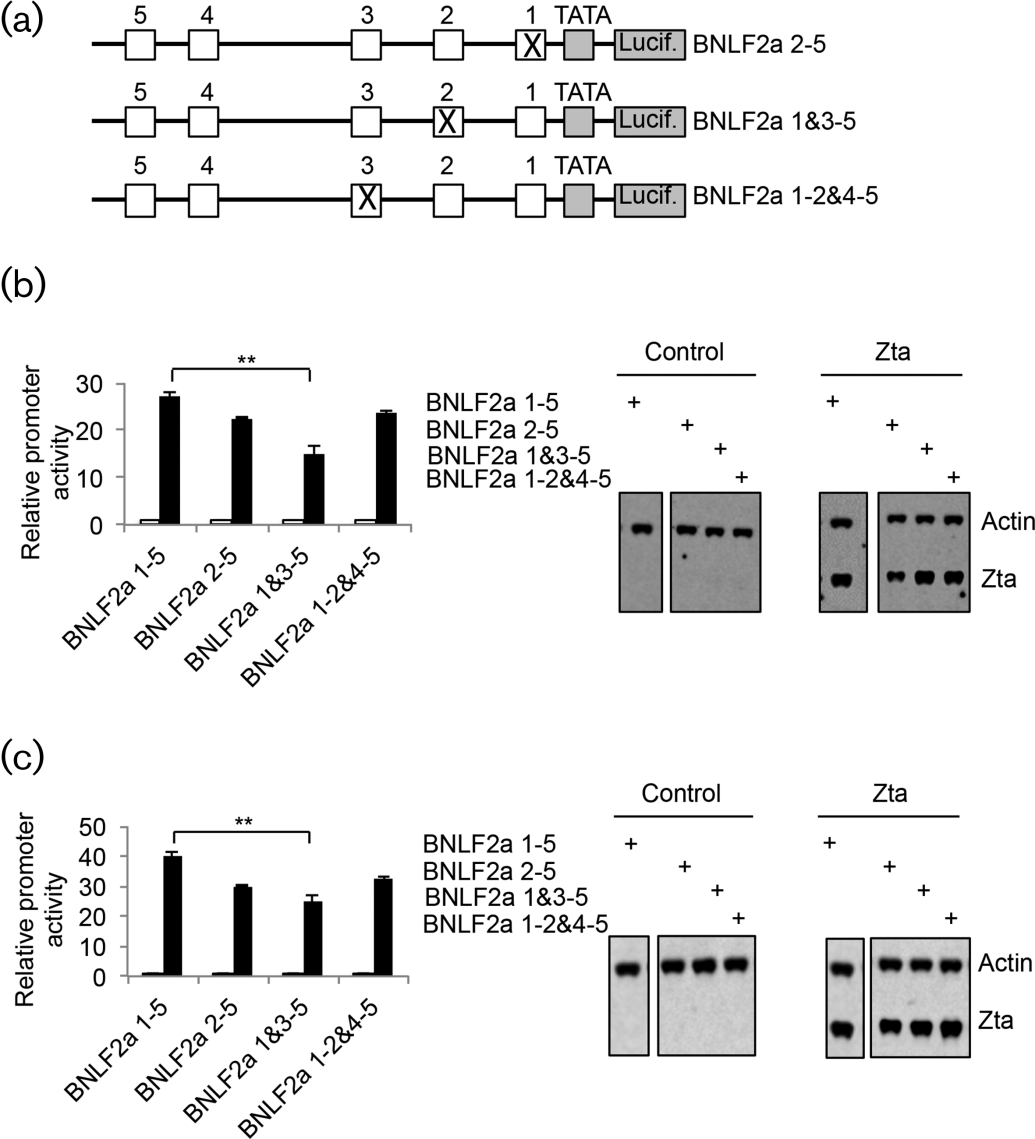
Contribution of individual proximal Zta-binding elements to *BNLF2a* promoter activation by Zta. (a). Schematic diagrams of the mutations introduced into the BNLF2a promoter–luciferase reporter system. (b). The indicated plasmids were introduced into DG75 cells with or without the his-Zta expression vector. Cells were incubated for 48 h and Zta-driven promoter activity determined. Zta and actin protein expression were determined following Western blotting. The significance of the difference in promoter activity between BNLF2a ZRE1, 3 and 5 and 1–5 is shown, **P≤0.01). (c). The indicated plasmids were introduced into HeLa cells with or without the his-Zta expression vector. Cells were incubated for 48 h and Zta-driven promoter activity determined as in (b).

To probe the contributions of the proximal ZREs further, individual mutations of ZRE1-3 were undertaken (Fig. 5). As shown in Fig. S4, small changes in basal expression resulted from these mutations. The ability of Zta to activate these promoters was assessed in both DG75 (Fig. 5b) and HeLa cells (Fig. 5c). This revealed that mutating either ZRE1 (BNLF2a 2–5) or ZRE3 (BNLF2a 1–2 and 4–5) alone did not have a significant impact on Zta activation, whereas mutating ZRE2 (BNLF2a 1 and 3–5) resulted in a modest but significant (*P*≤0.01) reduction in Zta activation to between 50–67 % of the wild-type promoter (BNLF2a).

### Impact of methylation on the BNLF2a promoter

**Fig. 6.**
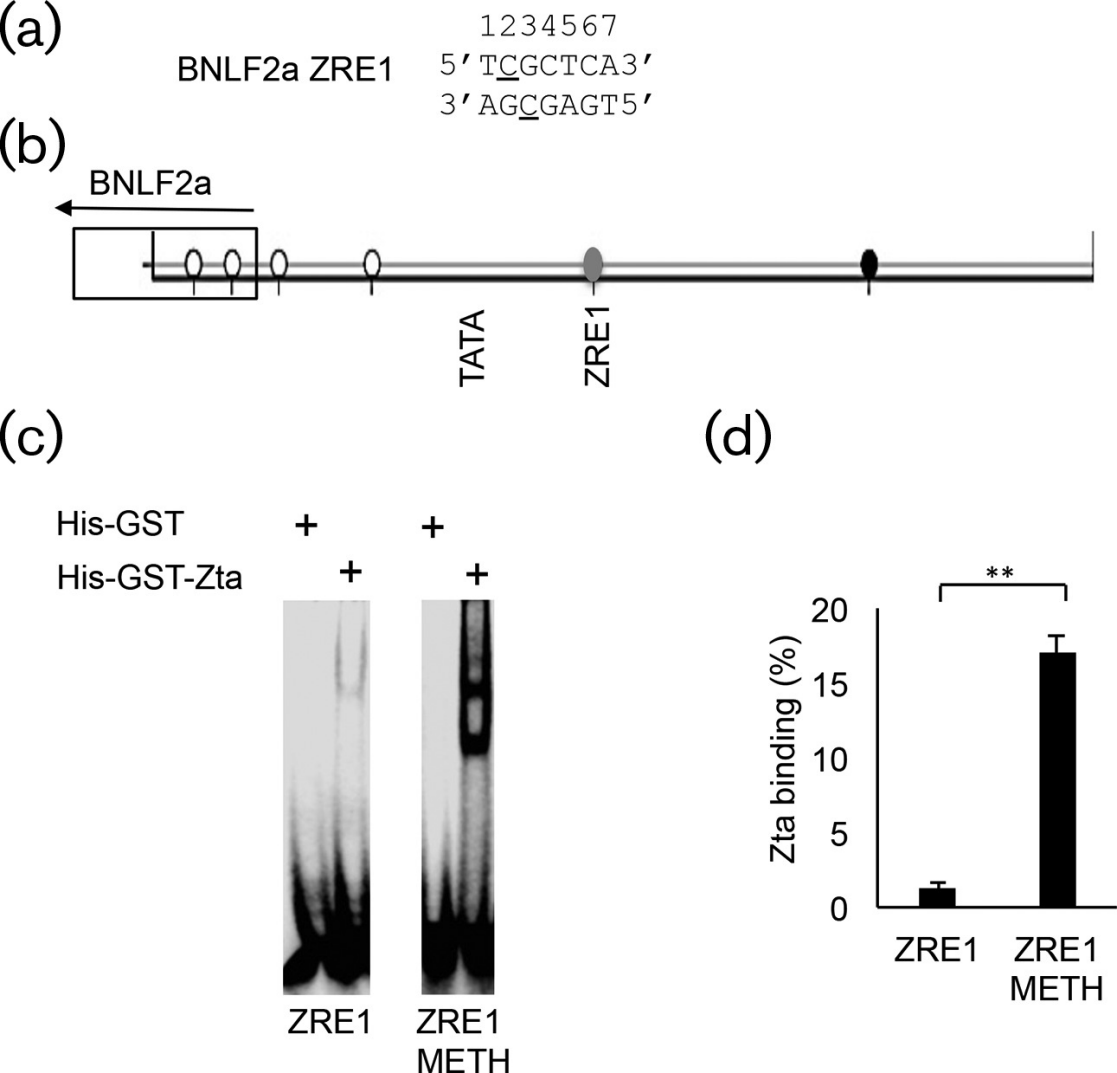
Methylation at BNLF2a ZRE1 and impact on Zta DNA binding (a). The DNA sequence of BNLF2 ZRE1 is shown with the C of each CpG motif underlined. (b). DNA methylation analysis of the 193 bp region surrounding the BNLF2a ZRE1 region of EBV is shown for Akata cell latency. The location and orientation of the BNLF2a coding region is indicated. Following bisulphite treatment of genomic EBV DNA, and amplification of the 193 base-pair region, the DNA was sub-cloned and individual colonies were subject to DNA sequence analysis. The methylation status of the six CpG motifs within the amplicon was determined and is shown. Black represents 100 % methylation, white represents non-methylation and grey represents a mixture of methylated and non-methylated templates. The locations of the TATA box and ZRE1 are shown for reference. (c). His-GST or His-GST-Zta was incubated with IR-labelled double-strand oligonucleotide probes corresponding to either *BNLF2a* ZRE1 or methylated ZRE1, and then the reactions were separated on native polyacrylamide gels using EMSA. The bar graph shows quantitation of binding (% of probe bound), together with the standard deviation from triplicate assays.

ZRE1 contains an integral CpG motif (Fig. 6a), which prompted us to ask whether the site is subject to DNA methylation in latency. We undertook DNA methylation sequencing in latent Akata cells. Following bis-sulphite conversion and desulphonation of genomic DNA, the region spanning ZRE1 of the *BNLF2a* promoter was amplified by PCR and clones captured in a pCDNA3 vector. Individual analysis of full-length clones that passed quality control checks showed that ZRE1 was subject to DNA methylation in a proportion of the clones (20 %) (Fig. 6b).

We then asked whether methylation of ZRE1 impacted on *in vitro* DNA binding. This was undertaken with the his-GST-Zta comparing to his-GST as a control (Fig. 6c). This revealed that methylation of the CpG motif within ZRE1 increased DNA binding significantly (*P*≤0.01).

**Fig. 7.**
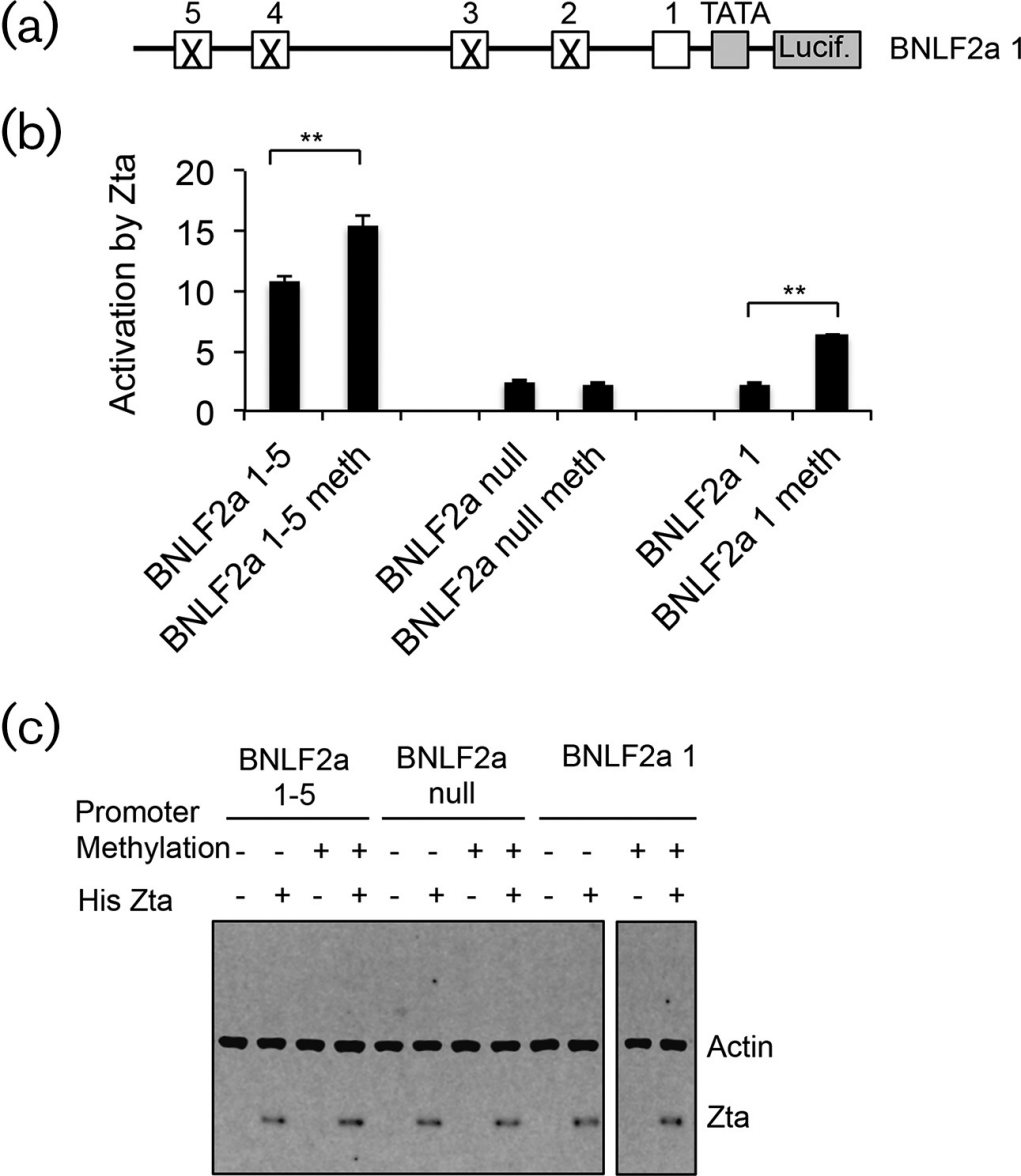
Impact of methylation of ZRE1 on *BNLF2a* promoter activity (a). Schematic diagram of mutations that leave only the proximal ZRE within the BNLF2a promoter. (b). The indicated plasmids were subject to methylation or not using CpG methyltransferase (M.SssI). These were introduced into 293T cells with either the his-Zta expression vector or its control vector. Cells were incubated for 24 h and promoter activity determined using luciferase assays. The black bars denote the change in promoter activity when his-Zta is expressed. The statistical significance of the difference in Zta-driven promoter activity between non- and methylated BNLF2a 1 is shown (**P≤0.01). (c). Zta and actin protein expression was determined following Western blotting.

The previous promoter assays were undertaken on non-methylated DNA. In order to determine whether DNA methylation had an impact on Zta-mediated activation of BNLF2a, we undertook *in vitro* DNA methylation of BNLF2a 1–5 and control promoters. Methylated and non-methylated promoters were then introduced into 293T cells with an expression vector for hisZta (Fig. 7). Little change in basal activity was observed (Fig. S6c). A modest but significant increase (*P*≤0.01) in activation by Zta was found when the BNLF2a 1–5 promoter was subject to DNA methylation (Fig. 7). In contrast, no activation was observed with the BNLF2a null promoter in either its methylated or non-methylated form. This activation is significant ((*P*≤0.01). To probe the relevance of ZRE1 for the enhanced methylation-dependent Zta activation of BNLF2a, we mutated ZRE 2–5 within the BNLF2a promoter, leaving only ZRE1 intact (BNLF2a 1) (Fig. 7). Following introduction into 293T cells, we found little variation in basal expression levels (Fig. S6c) and little activation of the non-methylated promoter by Zta. In contrast, we found a three-fold increase (*P*≤0.01) in Zta-driven promoter activity when the BNLF2a 1 promoter was methylated. In these experiments, the transfection efficiency differed only by 10 %.

## Discussion

*BNLF2a* is an important component of the programme used by EBV to evade the host immune response during the EBV lytic cycle and immediately following infection of B cells [22, 38, 39]. The 60-amino acid protein that it encodes blocks TAP presentation of antigens and so renders cells less susceptible to CD8+ recognition and subsequent destruction. Two other EBV genes, *BILF1* [40–42] and *BALF5* [43–45], have distinct roles in immune evasion from CD8+ cells. Indeed, within LCLs undergoing the spontaneous lytic cycle, it was recently shown that knockdown of the expression of *BGLF5* plays only a minor role in preventing antigen recognition by CD8+ cells, while in contrast knockdown of the expression of either *BNLF2a* or *BILF1* prevents antigen recognition [46]. *BNLF2a* is particularly influential in preventing recognition of epitopes derived from Zta and Rta, the earliest proteins expressed during EBV lytic cycle reactivation, whereas BILF1 protects against EBV epitopes expressed later during the cycle [46]. Together, these genes reduce CD8+-mediated destruction of cells harbouring EBV undergoing replication. If *BNLF2a* was not coordinately induced with the start of the EBV lytic cycle, then its expression and protection from CD8+ cells would not be coordinated with the onset of the lytic cycle and the production of infectious virus would be considerably diminished.

The expression of *BNLF2a* can also be regulated independently from EBV gene expression. For example, the promoter controls the expression of transgenes in a lineage-dependent manner driving high expression in stratifying epithelia – specifically the tongue, oesophagus and stomach [47, 48]. In addition, *BNLF2a* expression has also been characterized in gastric cancer in EBV-positive cells not undergoing the full programme of lytic cycle gene expression [49].

Here we identify *BNLF2a* as a direct target of the EBV lytic cycle regulatory protein Zta. This suggests that because Zta drives the expression of highly immunogenic lytic cycle proteins, it coordinately drives the expression of a viral immune evasion gene. This model is based on our discovery of two specific areas of interaction of Zta with the viral genome within the *BNLF2a* promoter. We established that the *BNLF2a* promoter is highly conserved between viral isolates, and we identified five conserved Zta-response elements within this region using *in silico* analysis and *in vitro* DNA binding assays. Critically, we also discovered that activation of the *BNLF2a* promoter in cells is dependent on these ZREs.

By analysing the requirements for Zta*-*mediated activation of the BNLF2a promoter in EBV-negative B cells and epithelial cells, we determined that Zta is able to act independently of other viral proteins to activate *BNLF2a*. However, the activation of certain EBV lytic cycle promoters is mediated by the synergistic action of the viral proteins Zta and Rta [16]. For example, BMRF1 (pEA-D) shows synergistic activation by Zta and Rta in certain cell lines but not in others [50, 51]. We therefore undertook a preliminary analysis to determine whether Rta activates or cooperates with Zta to synergistically activate the *BNLF2a* promoter, and to date have found no evidence in support of a role for Rta (data not show), although we cannot rule out the possibility that other EBV genes may contribute to regulation of the *BNLF2a* promoter.

Although the major impact of Zta-mediated activation of *BNLF2a* is driven through the proximal cluster of ZREs, our mutational analyses revealed that no single ZRE was able to effect full Zta-mediated activation (data not shown), and also that no single mutation at a ZRE prevented activation (Fig. 5). This suggests that more than one Zta binding event in the proximal promoter region of *BNLF2a* is required for *BNLF2a* activation, but that this can occur at any two of the three ZREs. This resonates with previous analyses of artificial promoter constructs. For example, Carey and colleagues constructed an artificial promoter using basic elements of the adenovirus E4 promoter abutted to varying numbers of ZREs [52], whereas Sato and colleagues converted the unresponsive promoter for the cellular collagenase gene into an Zta target by adding an additional ZRE [53]. As Zta contacts components of the basal transcriptional machinery (TFIIA and TFIID) to stabilize transcription complex assembly [54], these data support a simple model whereby as more than one molecule of Zta associates with a promoter, the transcriptional activation increases.

The majority of the EBV genome encoding lytic cycle genes is subject to DNA methylation at CpG motifs during latency [11, 29, 55, 56]. The *BNLF2a* promoter is located between the promoters LMP2a and LMP1. The broad methylation-mapping data from this region undertaken by Fernandez *et al.* gave a mixed picture, showing promoter-specific DNA methylation in certain samples of EBV infected cells but not in others [55]. We decided to ask the specific question of whether the integral CpG motif within ZRE1 of the *BNLF2a* promoter is subject to DNA methylation during EBV latency in Akata BL cells. Our analysis revealed that DNA methylation at this CpG motif occurs, but that it is not present in all of the genome copies. This is broad in agreement with Fernandez *et al*. Importantly, we show that DNA methylation of CpG within ZRE1 increases the ability of Zta to interact with ZRE1 and that *BNLF2a* promoter activation can be driven through a methylated *BNLF2a* promoter when ZRE1 is the only ZRE present. Methylation-dependent Zta activation was less than the 100-fold-change that has been reported for another EBV promoter (Na); this is almost fully CpG methylated, contains two CpG-ZREs and displayed a 100-fold change in Zta mediated activation [57]. However, as *BNLF2a* is only 20 % methylated and has only one CpG ZRE, this is not surprising. Nonetheless, as ZRE1 is not sufficient to drive full promoter activity, this suggests that interaction of Zta with more than one ZRE is required for optimal activation of even the methylated *BNLF2a* promoter.

The question of what drives the activation of BNLF2a during primary infection of B cells remains unanswered. There is evidence that Zta RNA is expressed at this stage of the pre-latency cycle, but no evidence has been found for the protein [3, 22]. So, while it may be tempting to speculate that the coincident expression of both Zta and *BNLF2a* suggests that Zta drives *BNLF2a* in this situation, there is no evidence that supports this [3]. It could also be speculated that cellular transcription factors are responsible for this – as is likely to be the case in gastric cancer cells [49].

In summary, we have demonstrated that Zta can directly regulate *BNLF2a*. However, it is clear that a simple model to explain the regulation by Zta will not suffice. There is no single element required to drive full activation – both the epigenetic status of a single CpG motif with the promoter and a redundancy in Zta binding sites in the proximal promoter region contribute to Zta-driven promoter activity. We speculate that the redundancy of ZREs within the promoter provides a fail-safe mechanism for Zta-mediated *BNLF2a* activation, ensuring that if access to one ZRE is blocked, perhaps through interaction with a cellular factor, Zta interaction with the other two sites will allow it to activate this important promoter.

## Methods

### Cell culture and transfection

Group I EBV-positive Akata BL cells [58], Raji cells [59], the lymphobalastoid cell lines LCL#3 [32] and GM12878, and DG75 EBV-negative Burkitt Lymphoma cells [60], were maintained in RPMI medium supplemented with 10 % (v/v) fetal bovine serum, 100 U of penicillin/ml, 100 µg of streptomycin ml^−1^ and 2 mM l-glutamine (Invitrogen) at 37 °C with 5 % (v/v)CO_2_. For EBV lytic induction of Akata cells with anti-IgG, cells were seeded in log-phase growth at 5×10^5^ cells ml^−1^. After 24 h, the cells were concentrated to 2×10^6^ cells ml^−1^ and treated with a low dose of 0.125 % (v/v) rabbit anti-human IgG (Dako) or Dulbecco phosphate-buffered saline for 48 h. As judged by intracellular FACS staining, using BZ1 monoclonal antibody the FIX and PERM Cell Permeabilization Kit and detected using FACS accuri (Beckton Dickinson), we found that 6 % of LCL#3 and 7 % of Akata cells were undergoing the lytic cycle (data not shown).

293T cells (ECACC 12022001) and HeLa cells (ECACC 93021013) were cultured in DMEM medium with 10 % (v/v) fetal bovine serum, 100 U of penicillin ml^−1^, 100 µg of streptomycin ml^−1^ and 2 mM l-glutamine (Invitrogen) at 37 °C with 5 % (v/v) CO_2_.

Transfections into lymphocytes were undertaken using electroporation with a total of 10 µg of DNA per 10^7^ cells (50 : 50 expression vector: luciferase reporter) in 300 µl serum-free media. A 4 mm gap-size cuvette was used with a Genepulser II (BIORAD) set at 250 volts and 975 ohms capacitance. Cells were harvested 24 h later. Transfections into adherent cells were undertaken using the non-liposomal effectene reagent (Qiagen). Cells were seeded in 6-well plates at 4×10^5^ cells/well. The cells were transfected with a total of 1 µg of DNA (500 ng expression vector DNA with 500 ng reporter vector DNA). Effectene was used at a ratio of 2.5 µl per µg DNA. Cells were harvested 48 h later, lysed and processed using the Firefly luciferase assay system (Promega), with protein concentration determined using a Bradford or BCA assay (Biorad). Total protein extracts were also prepared by lysis in SDS-PAGE sample buffer, and Zta and actin abundance monitored using Western blot analysis. The promoter data were normalized to the total protein concentration or the actin signal. Promoter activity from triplicate assays was determined and all experiments were undertaken on at least two occasions.

Intracellular FACS staining, using BZ1 monoclonal antibody and FIX and PERM Cell Permeabilization Kit (*Invitrogen*), was used to determine the population of cells in the lytic cycle.

### Chromatin precipitation

Chromatin was prepared as described previously [19, 61]. Trimethylated histone H3K9 (Abcam ab8898) and trimethyl histone H3K27 (Abcam ab6002) and species-specific controls were used for the Histone modification chromatin precipitation assays at the BNLF2a, BRLF1, Cp, Qp and GAPDH promoters and OriLyt. Goat polyclonal antibody (sc-17503) against Zta or a control goat antibody (Santa Cruz Biotechnology) was used for the chromatin precipitation for Zta. The Q-PCR primer sets were published previously [7, 18, 19].

### Western blot

Western blotting. Total cell lysates were resolved on a 12 % (w/v) Bis-Tris Nu- PAGE gel in morpholine propanesulfonic acid buffer (Invitrogen). After SDS-PAGE, the proteins were transferred onto nitrocellulose membranes (Santa Cruz Biotechnology) and incubated with indicated antibodies overnight at 4 °C. BZ1 mouse monoclonal antibody to Zta [14] and a rabbit antibody to beta actin (Sigma) were used to detect proteins by Western blotting. IR-labelled anti-mouse and IR-labelled anti-rabbit (Licor) were used as secondary antibodies and the signals were detected using infrared detection (Licor Odyssey).

### Promoter constructs and plasmids

A cDNA3-based expression vector for Zta that includes a hexa-histidine tag at the amino terminus (hisZta), together with its control vector, was used [62]. The *BNLF2a* promoter was cloned into the pCpGL-basic expression vector using BamHI and HindIII [63]. The sequence coordinates for the *BNLF2a* promoter are 167029–167941 referring to the Human herpesvirus 4 type 1, complete genome NCBI Reference Sequence: NC_007605.1. The TATA box is located between 167062 and 167067. A BamHI site was introduced at the distal and a HindIII site at the promoter proximal end using gene synthesis. For each of the ZRE mutations the ZRE was mutated to CCCCTTT (distal to proximal). For DNA methylation experiments, the relevant plasmids were incubated with or without CpG methyltransferase (M.SssI) in the presence of 160 µM S-adenosylmethionine for 1 h at 37 °C as recommended by the manufacturer (New England Biolabs), then purified using a plasmid mini clean-up column (Qiagen).

The His-tagged GST-Zta expression vector was generated by cloning the coding region for Zta (aa 168–245) into the pOPINJ vector [a gift from Ray Owens (Addgene plasmid #26045)].

### Methylation sequencing

Akata cells were subjected to acyclovir treatment for 48 h to suppress EBV replication [7]. DNA was prepared using a Qiamp kit (Qiagen), and 800 ng of genomic DNA was subject to C to T conversion according to the EZ DNA methylation kit (Zymogen) essentially as described [64]. Nucleotides 166500–166800 of the Akata virus (KC207813.1 Human herpesvirus 4 strain Akata), surrounding BNLF2a ZRE1, were used to design amplification primers using the following parameters: primer length 24–38, product length 100–350, Tm 55–65 and 1 CpG in first 1/3 of primer. The chosen primers were TTAATTTATGTTAGTAGAGGTAGGAATATTTGTTG and AACAAACCRCAAACAAAAAACTACT ACTCTAACAAAAC. The expected 266 nucleotide amplicon contained one CpG within the primer and five within the sequence. BamH1 and EcoR1 cloning sites were added to the 5′ ends of the primers, together with a six-nucleotide clamp CCCGGC sequence at the 5′end. Amplification of 25 % of the converted DNA was undertaken with EpiTaq (TaKaRa) using a gradient 2.00, 2.25, 2.50, 2.75 and 3.00 mM MgCl_2_, and 40 cycles of 10 s 95 °C, 10 s 52.5 °C, 30 s 72 °C. Amplification was successfully detected using both 2.75 mM and 3.00 mM MgCl_2_. DNA was isolated using the Qiaquick PCR purification kit (Qiagen) and subject to restriction digestion with EcoR1 and BamH1 (NEB). The cloning vector pcDNA3 (Invitrogen) was also subject to digestion with EcoR1 and BamH1 (NEB). Following ligation and transformation into *E. coli*, individual colonies were isolated and subject to DNA sequencing using the T7 primer. Clones with a full-length insert quality controlled (% conversion ≥95 %) using BISMA [65] and the percentage conversion at each CpG locus were determined. This was plotted graphically using the tool methylation plotter [66].

### *In vitro* DNA binding assays

His-tagged GST-Zta (His-GST-Zta) was expressed in Rosetta pLysS *E. coli* in the presence of ampicillin and chloramphenicol. Expression was induced after transferring the culture to auto-induction media (Overnight Express Instant TB Medium, Novagen) and incubating at 25 °C for 20 h. The cell pellet was lysed by one round of freeze–thaw followed by extraction in 50 mM Tris HCl pH 7.5, 500 mM NaCl, 0.5 mM TCEP, 1.5 ul ml^−1^ benzonase and 0.2 % Tween. The soluble protein was loaded onto a Talon cobalt affinity column (GE) coupled to an AKTA FPLC (GE) and subsequently eluted using 50 mM Tris HCl pH7.5, 500 mM NaCl, 0.5 mM TCEP and 150 mM imidazole. The eluted protein was concentrated four-fold using Ultra-4 centrifugal filters (Amicon) with a 3Kd molecular weight cut-off. Subsequently, 500 ul of the sample was loaded onto a GF 75 size exclusion chromatography column (GE) and eluted using 50 mM Tris HCl, 300 mM NaCl and 0.5 mM TCEP. A recombinant His-GST protein (Abcam) was compared as control.

IR-800-labelled double-strand DNA oligonucleotides relating to each of the five *BNLF2a* ZREs, a mutant version of ZRE2 and a CpG-methylated version of ZRE1 were annealed (IDT) (Table 1). Protein (100 ng) was incubated with the double-strand oligonucleotides probe (1 nmol) in a total of 20 µl of gel shift buffer (4 % (w/v) glycerol, 1 mM MgCl, 0.5 mM EDTA, 5 mM DTT, 50 mM NaCl and 10 mM Tris-HCl (pH 7.5) for 30 min at 20^ °^C. Following separation on a 6 % (w/v) native polyacrylamide gel (0.5XTBE), for 1 h at 10 V cm^−1^, fluorescent signals were detected in the IR Odyssey imager (LI-COR) in the 800 channel. Quantitation was undertaken using Image Studio software (Licor).

**Table 1.** Oligonucleotides (forward and reverse strands) used as probes for electrophoretic mobility shift assays. The ZREs is shown in bold and underlined

DNA probe (**5**' to **3'**)	DNA sequence	EBV coordinates NC_007605.1
BNLF2a ZRE1 F	ACACCTGTCC **TCGCTCA** TCTTTCCACA	167099 – 167125
BNLF2a ZRE1 R	TGTGGAAAGA **TGAGCGA** GGACAGGTGT	
BNLF2a ZRE2 F	CACCTGTTGT **TGACACA** TTCTTTGCGC	167183 – 167209
BNLF2a ZRE2 R	GCGCAAAGAA **TGTGTCA** ACAACAGGTG	
BNLF2a ZRE3 F	CTTTCCATCT **TGTGCCA** ATACACATTT	167256 – 167282
BNLF2a ZRE3 R	AAATGTGTAT **TGGCACA** AGATGGAAAG	
BNLF2a ZRE4 F	TCACCTTAAC **TGGCACA** CACTCCCTTA	167593 – 167619
BNLF2a ZRE4 R	TAAGGGAGTG **TGTGCCA** GTTAAGGTGA	
BNLF2a ZRE5 F	TAAGCTACTA **TGACTAA** CCTTTCTTTA	167692 – 167718
BNLF2a ZRE5 R	TAAAGAAAGG **TTAGTCA** TAGTAGCTTA	
BNLF2a ZRE1meF	ACACCTGTCC **TmeCGCTCA** TCTTTCCACA	167099 – 167125
BNLF2a ZRE1me R	TGTGGAAAGA **TGAGmeCGA** GGACAGGTGT	

## Supplementary Data

1860 Supplementary File 1
