# Supplementary material for: Mechanism of activation of the BNLF2a immune evasion gene of Epstein-Barr virus by Zta
Source: J Gen Virol. 2018 Mar 26;99(6):805–17. doi: 10.1099/jgv.0.001056 (PMC6096924; doi:10.1099/jgv.0.001056)
Supplement: Supplementary File 1 [file jgv-99-805-s001.pdf]

**Supplementary information**

# Mechanism of activation of the *BNLF2a* immune evasion gene of Epstein-Barr virus by Zta

Rajaei Almohammed[a], Kay Osborn[a], Sharada Ramasubramanyan[a*], Ijiel Barak Naranjo Perez-Fernandez[a], Anja Godfrey[a], Erika J. Mancini[a], Alison J. Sinclair[a#].

## *Methods for identification of potential ZREs*

The location of potential ZREs was undertaken using the ZRE prediction tool as described previously (Flower *et al.*, 2011). *BNLF2a* promoter sequences were identified from NCBI nucleotide databank using the basic local alignment search tool (Blast) (Camacho *et al.*, 2009) to identify all alignments with the nucleotide collection containing 29,967,113 non-redundant entries from GenBank, EMBL, DDBJ, PDB and RefSeq. 92 sequences from whole genome assemblies were selected and aligned using Clustal Omega (Sievers & Higgins, 2014; Sievers *et al.*, 2011) and viewed using JalView (Clamp *et al.*, 2004). The 92 genome entries and sequence co-ordinates are shown in Supplementary Table 1. The DNA sequence motif describing conservation of nucleotides within the 5 ZREs was generated using MEME (Bailey *et al.*, 2015).

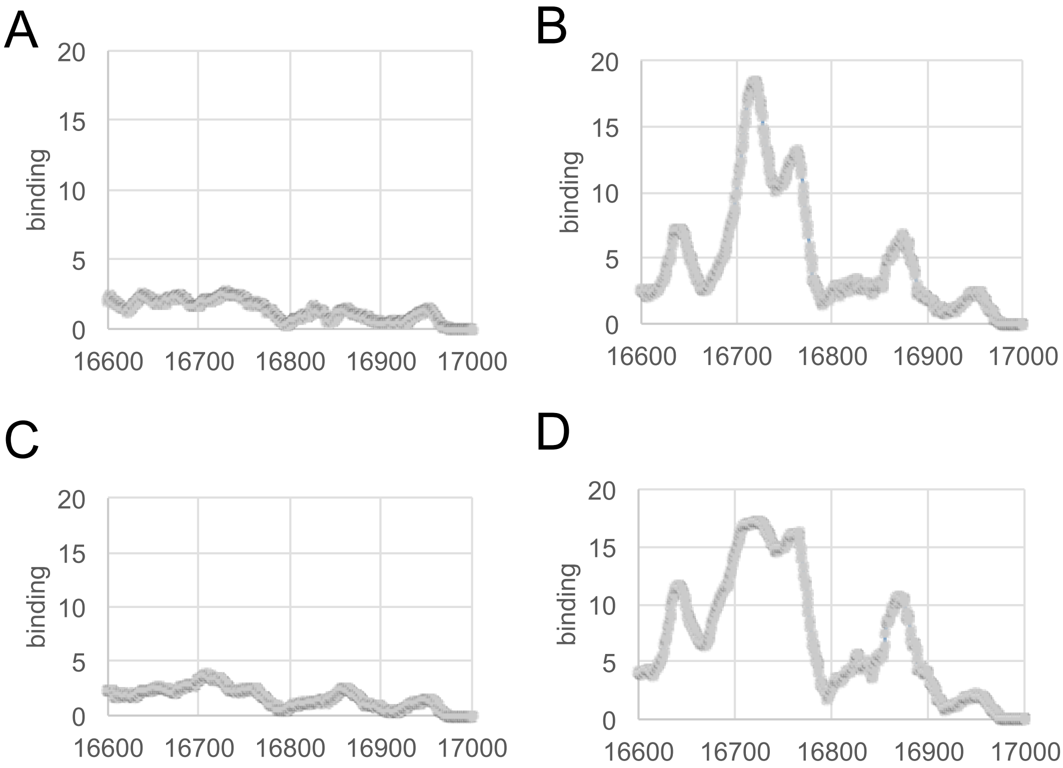


**Supplementary Figure 1. Control ChIP experiments comparing input, with Zta binding in early and late lytic cycle.**

**Supplementary Figure 1. Control CHIP experiments comparing input, with Zta binding in early and late lytic cycle.**

ChIP-sequencing undertaken in lytic cycle induced Akata cells is presented in the BNLF2a promoter region (data from study by (Ramasubramanyan *et al.*, 2015)). The EBV genome co-ordinates are shown on the X-axis and the raw sequence reads on y-axis “binding”.

A. The signal from early lytic cycle input chromatin is shown.

B. The signal from the Zta-chromatin immunoprecipitation in early lytic cycle is shown.

C. The signal from full lytic cycle input chromatin is shown.

D. The signal from the Zta-chromatin immunoprecipitation in full lytic cycle is shown.

A

GGATCCCATAGCCCTAGCGACTCTGCTGGAAATGATGGAGGCCCTCCACAATTGACGGAA
GAGGTTGAAAACAAAGGAGGTGACCAGGGCCCGCCTTTGATGACAGACGGAGGCGGCGGT
CATAGTCATGATTCCGGCCATGGCGGCGGTGATCCACACCTTCCTACGCTGCTTTTGGGT
TCTTCTGGTTCCGGTGGAGATGATGACGACCCCCACGGCCCAGTTCAGCTAAGCTACTA**T**
**GACTAA**CCTTTCTTTACTTCTAGGCATTACCATGTCATAGGCTTGCCTGACTGACTCTCC
CTCCATTTACTGGGAATGCCTTAGCTAATCACCTTAAC**TGGCACA**CACTCCCTTAGCCAC
ACTGTCTGTCTAGGCTGAAAAGCCACATTCATATTCTATTTCAAAACAAGGGGAAAGGAG
GACATGCGAGAATTGGCAGACACCTTTACCCAGCCCTTAACACACCACACAGGTAGCAAG
GACCCGGGCGTTGCCAGACTCCGCCACCAACGCCCCTGCGTTGAACCCACCCCTCCTACA
CACATCAGACCTCTGCACAACACAACTACCAGGCAGATGAGGCCCCTTACTTCCACAGGG
TACTGGCATACCAGCGGGGGACCACATACATCCCTGTCTCCCACCCAGTAACTCCAGCAA
CTTTGCTTTCCATCT**TGTGCCA**ATACACATTTGGATTCAGCCCAAGCCACACCTAACTCA
TGCCAGCAGAGGCAGGAACACCTGTTGT**TGACACA**TTCTTTGCGCATAAGCACTTTAATC
CCTCTCTCACACCCAGAAACTAAGAGCTAGCCCAAAACCTCCACACCTGTCC**TCGCTCA**T
CTTTCCACATTCCTCTGGCCTTCTTTCCTTGTCCTTACTG*TATAAA*AGTCCACGAAAACA
GCTGTGCCTCACTCTCGAGCTCGAG

B

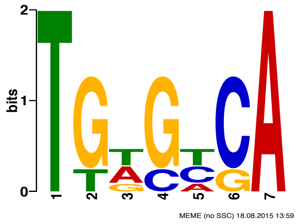


C

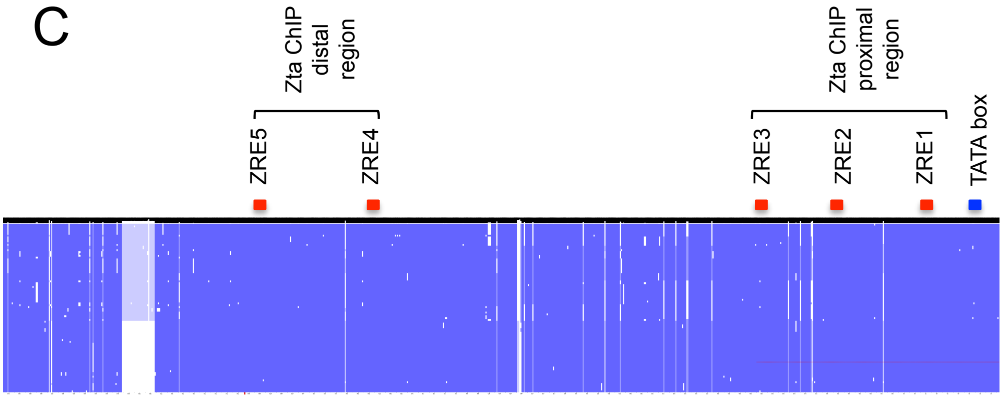


**Supplementary Figure 2. The location of ZREs within the *BNLF2a* promoter.**

**Supplementary Figure 2. The location of ZREs within the *BNLF2a* promoter.**

A. Five potential ZREs were identified within the EBV genome that contains the BNLF2a promoter (930bp) using an established algorithm based on 32 different ZRE sequences. The ZREs are shown in bold and underlined and the location of the TATA box in italic and underlined.

B. The conserved sequence motif representing the five predicted ZREs was identified using the motif-based sequence analysis tool considering the seven nucleotide ZREs (MEME).

Sequence variants within the 930-nucleotide region of the BNLF2a promoter from 92 EBV genomes (supplementary Table 1) were aligned (Clustal Omega version 1.2.4) and then viewed with Jalview. The degree of DNA sequence consensus is indicated by the upper black box. The location of the potential ZREs, the TATA box and the Zta ChIP-peaks are shown above the aligned sequences. The blue area represents the 92 sequences at each of nucleotide position; homologous sequences are shown in blue and non-homologous as white blocks. The database codes for the sequences used are shown in Supplementary table 1.

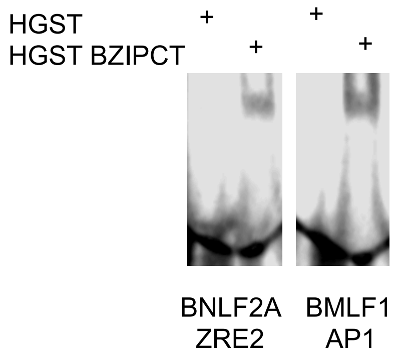


**Supplementary Figure 3. Comparison of interaction of His-GST-Zta with BNLF2a ZRE2 and a known ZRE (BMLF1 ZRE)**

**Supplementary Figure 3. Comparison of interaction of His-GST-Zta with BNLF2a ZRE2 and a known ZRE (BMLF1 ZRE)**

80ng of His GST and His-GST-Zta proteins were incubated with IR-labeled double strand oligonucleotide probes corresponding to BNLF2a ZRE2 or a the API site from the BMLF1 promoter. The reactions were then separated on native

polyacrylamide gels using EMSA. The migration of free DNA and bound DNA complexes are shown.

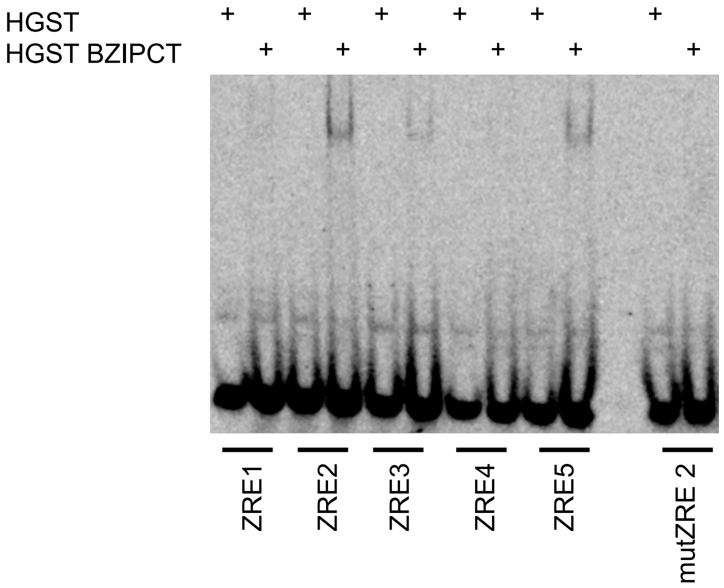


**Supplementary Figure 4. Comparison of interaction of His-GST-Zta withall five BNLF2a ZREs**

**Supplementary Figure 4. Comparison of interaction of His-GST-Zta with all five BNLF2a ZREs**

80ng of His GST and His-GST-Zta proteins were incubated with IR-labeled double strand oligonucleotide probes corresponding to each BNLF2a or a mutant ZRE2 site. The reactions were then separated on native polyacrylamide gels using EMSA. The migration of free DNA and bound DNA complexes are shown.

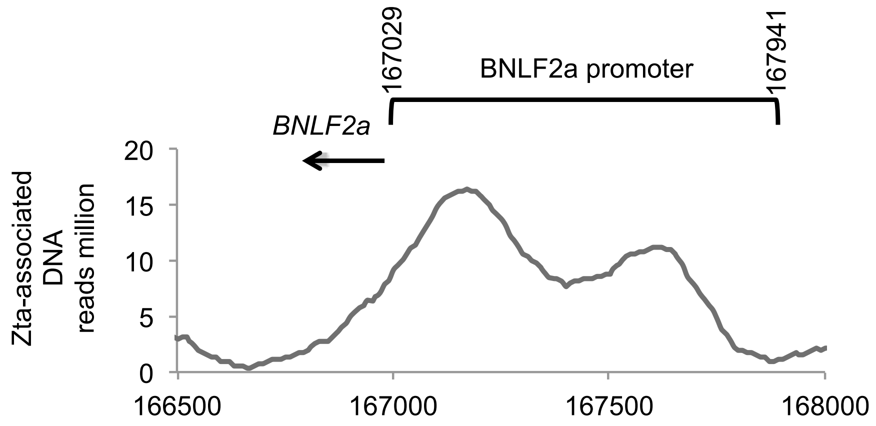


**Supplementary Figure 5. The location of region upstream from *BNLF2a* chosen for promoter analysis.**

**Supplementary Figure 5. The location of region upstream from *BNLF2a* chosen for promoter analysis.**
ChIP-sequencing undertaken in lytic cycle induced Akata cells is presented in the BNLF2a promoter region (data from study by (Ramasubramanyan *et al.*, 2015)). The EBV genome co-ordinates are shown on the X-axis and the background subtracted reads on y-axis. The location of the region analyzed as the *BNLF2a* promoter is indicated with the location and orientation of the *BNLF2a* coding region.

A. The signal from early lytic cycle input chromatin is shown.

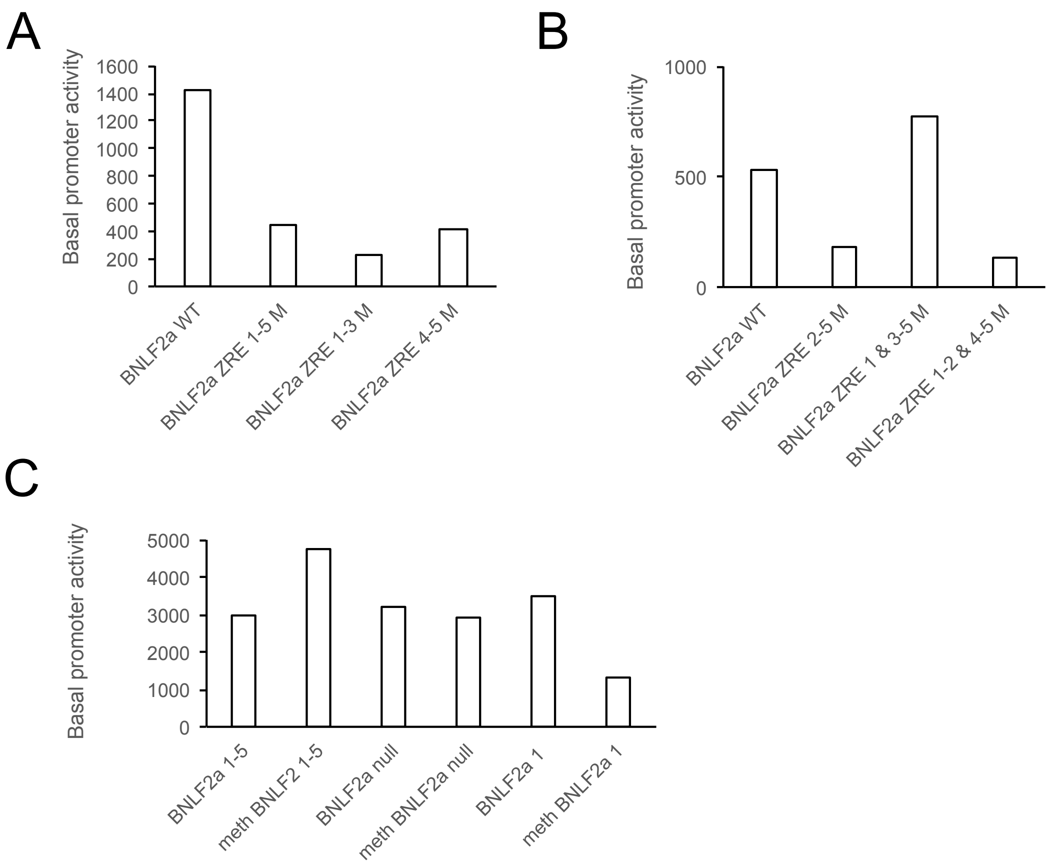


**Supplementary Figure 6. The basal promoter activity of promoter constructs used DG75 (A and B) and 293T (C)**

**Supplementary Figure 6. The basal promoter activity of promoter constructs used.**
The indicated plasmids were introduced into cells without the His-Zta expression vector. Cells were incubated for 48 hours and basal promoter activity determined. DG75 cells (A and B) or 293T cells (C) were used.

| **Table 1. EBV isolate sequences in BNLF2a promoter region** | |
|---|---|
| **Genbank entry** | **sequence co-ordinates** |
| **LN827527.1** | **167162-168074** |
| **LN827593.1** | **167171-168083** |
| **LN827583.1** | **167286-168198** |
| **LN827575.1** | **167355-168267** |
| **KC440852.1** | **167041-167953** |
| **LN827588.1** | **167212-168124** |
| **LN827580.1** | **167406-168318** |
| **LN827557.2** | **167164-168076** |

| | |
|---|---|
| **LN827548.2** | **167289-168201** |
| **LN827800.1** | **167348-168260** |
| **LN827582.1** | **167133-168045** |
| **LN827556.1** | **167474-168386** |
| **LN827544.1** | **167239-168151** |
| **LN827577.1** | **167122-168034** |
| **LN827545.1** | **167308-168220** |
| **LN827565.1** | **167096-168008** |
| **LN827573.1** | **167130-168042** |
| **LN827571.1** | **167127-168039** |
| **LN827566.1** | **167127-168039** |
| **LN827558.1** | **167129-168041** |
| **LN827552.1** | **167121-168033** |
| **LN827550.1** | **167132-168044** |
| **KC207814.1** | **166889-167801** |
| **LN827585.1** | **167127-168039** |
| **KF717093.1** | **161382-162294** |
| **AB828191.1** | **159694-160606** |
| **LN827569.1** | **167175-168087** |
| **LN827524.1** | **167220-168132** |
| **LN827553.1** | **167050-167962** |
| **LN827592.1** | **167173-168085** |
| **LN827595.1** | **166976-167888** |
| **LN827584.1** | **167058-167970** |
| **LN831023.1** | **167341-168225** |
| **LN827560.1** | **167469-168353** |
| **DQ279927.1** | **168513-169397** |
| **KC617875.1** | **166550-167431** |
| **LN827594.1** | **167139-168021** |
| **AB850658.1** | **165747-166629** |
| **LN824209.1** | **167267-168149** |
| **LN824208.1** | **167002-167884** |
| **KC207813.1** | **166570-167452** |
| **LN827554.1** | **160956-161838** |
| **LN827563.2** | **167114-167996** |
| **LN827591.1** | **167343-168225** |
| **AB850649.1** | **161667-162549** |
| **LN827525.1** | **167134-168016** |
| **AB828190.1** | **162948-163830** |
| **KJ411974.1** | **166396-167278** |
| **KF992567.1** | **166725-167607** |
| **KF992568.1** | **166903-167785** |

| | |
|---|---|
| LN827547.1 | 167254-168136 |
| LN824224.1 | 167250-168132 |
| AB850660.1 | 165302-166184 |
| AB850657.1 | 162705-163587 |
| AB850651.1 | 163507-164389 |
| KF992571.1 | 166390-167272 |
| KF992570.1 | 166698-167580 |
| KF992569.1 | 165728-166610 |
| KF992566.1 | 166596-167478 |
| KF992565.1 | 166647-167529 |
| KF992564.1 | 165439-166321 |
| JQ009376.2 | 166621-167503 |
| KF373730.1 | 167002-167884 |
| HQ020558.1 | 161522-162404 |
| AY519199.1 | 1191-2073 |
| LN824142.1 | 167046-167928 |
| LN827549.1 | 167115-167997 |
| AB850646.1 | 164166-165048 |
| AB850643.1 | 160975-161857 |
| AY961628.3 | 166940-167822 |
| LN827561.1 | 167112-167994 |
| LN827586.1 | 167160-168072 |
| LN827578.1 | 167443-168355 |
| LN827562.1 | 167144-168056 |
| LN827523.1 | 166830-167742 |
| LN827799.1 | 167155-168067 |
| LN827739.1 | 167082-167994 |
| LN827597.1 | 166966-167878 |
| LN827572.1 | 167175-168087 |
| LN827555.1 | 167018-167930 |
| AB850654.1 | 167030-167942 |
| AB850647.1 | 166872-167784 |
| V01555.2 | 167487-168399 |
| AJ507799.2 | 167029-167941 |
| LN827551.1 | 166920-167832 |
| LN824203.1 | 167048-167960 |
| LN827581.1 | 167138-168050 |
| LN827579.1 | 167013-167925 |
| LN827574.1 | 166992-167904 |
| LN827568.1 | 167121-168033 |
| LN824205.1 | 167130-168042 |

**Supplementary Table 1.**

| ZRE Name | 5'-3' Sequence | EBV (NC_007605.1) Coordinates | Mutated sequence |
|---|---|---|---|
| **ZRE1** | TCGCTCA | 167109-167115 | CCCCTTT |
| **ZRE2** | TGACACA | 167194-167199 | CCCCTTT |
| **ZRE3** | TGTGCCA | 167266-167272 | CCCCTTT |
| **ZRE4** | TGGCACA | 167609-167603 | CCCCTTT |
| **ZRE5** | TGACTAA | 167702-167708 | CCCCTTT |

**Supplementary Table 2.** Sequence and EBV co-ordinates of ZRE1-5 and the substitutions used in mutants affecting them.

| Plasmid name | ZREs mutated | ZREs remaining |
|---|---|---|
| BNLF2a 1-5 | none | ZRE1-5 |
| BNLF2a Null | ZRE1-5 | none |
| BNLF2a 1-3 | ZRE4-5 | ZRE1-3 |
| BNLF2a 4-5 | ZRE1-3 | ZRE4-5 |
| BNLF2a 2-5 | ZRE1 | ZRE2-5 |
| BNLF2a 1&3-5 | ZRE2 | ZRE1&3-5 |
| BNLF2a 1-2&4-5 | ZRE3 | ZRE 1-2&4-5 |
| BNLF2a 1 | ZRE2-5 | ZRE1 |

**Supplementary Table 3.** Promoter-reporter plasmid names and the details of ZRE mutations and intact remaining ZREs.
